# Oral pathology in a population observed within an oral cancer screening developed in Portugal

**DOI:** 10.4317/medoral.26863

**Published:** 2024-11-25

**Authors:** Diogo Pereira, Margarida Andrade, André Moreira, João Caramês, Marta Pojo, Filipe Freitas

**Affiliations:** 1 DDS. School of Dental Medicine, University of Lisbon, Lisbon, Portugal; 2 DDS, PhD. School of Dental Medicine, University of Lisbon, Lisbon, Portugal; 3 MSc, PhD. The Portuguese Cancer League, Lisbon, Portugal

## Abstract

**Background:**

Oral cancer is a global public health problem. Late diagnosis leads to treatment delays, which consequently result in a worse prognosis and a decrease in the 5-year survival rate. The purpose of this study is to evaluate the oral pathology in a population observed within an oral cancer screening developed in Portugal.

**Material and Methods:**

Oral cancer opportunistic screening activities were conducted by the non-profit organization Liga Portuguesa Contra o Cancro ‑ Núcleo Regional do Sul (LPCC-NRS) and data collection occurred between March and December 2022. Participants completed an anonymous questionnaire to collect sociodemographic data and lifestyle habits, followed by a clinical examination of the oral cavity. All individuals classified with oral potentially malignant disorders (OPMD), or suspected lesions of malignant neoplasia were subsequently contacted between January and February 2023 and were questioned about undergoing a biopsy and its respective pathological examination result. Descriptive and analytical statistics were applied.

**Results:**

A total of 2674 participants with a mean age of 57 years were screened. Fordyce granules (16.30%) were the most common non-pathological condition, and hairy tongue (11.04%) was the most frequently observed benign lesion. Leukoplakia (43.58%) was the most common oral potentially malignant disorder. Additionally, histological confirmation was obtained for the presence of 4 oral squamous cell carcinomas (OSCC), representing 0.15% of all population screened.

**Conclusions:**

Although benign pathology is the most frequently found, oral potentially malignant disorders and suspected malignant lesions were identified in 6.3% of participants in these oral cancer screenings. In this regard, we conclude that the oral cancer screening actions developed by LPCC-NRS were effective in identifying positive cases of OPMDs and oral cancer, using an intraoral visual examination of the oral cavity.

** Key words:**Oral cancer, oral potentially malignant disorders, oral cancer screening, oral pathology.

## Introduction

Oral cancer is a global public health issue, encompassing malignant tumors that affect the tissues of the oral cavity, lips, oropharynx, salivary glands, and maxillary bones. The most frequent malignant tumor in the head and neck is the oral squamous cell carcinoma (OSCC), comprising about 90% of all cases of oral cancer. This tumor originates in the epithelial tissues of the oral mucosa and, in most cases, is moderately to well-differentiated (1,2).

According to GLOBOCAN data released in 2020 by the International Agency for Research on Cancer (IARC), 431 300 new cases of oral cancer were diagnosed worldwide, with 377 700 cases of cancer of the lip, tongue, and oral cavity, and 53 600 cases of salivary gland cancer. Globally, the standardized incidence rates per 100 000 inhabitants, considering oral cancers are 6.7 in males and 2.8 in females (3).

In Portugal, according to the National Oncology Register of 2019, 1335 new cases of lip (C00), tongue (C01-02), oral cavity (C03-06), salivary glands (C07-08), tonsil (C09), and oropharynx and other cancers (C10) were diagnosed, with 1000 of those cases in men and 335 in women. The overall incidence rate was 13.0/100 000 inhabitants (20.7/100 000 in males and 6.2/100 000 in females). Additionally, oral cavity and oropharynx cancer rank as the sixth most frequent cancer in males. Portuguese age-standardized mortality rates for both sexes combined in 2019 were 3.3-4.3 for lip and oral cancer and were greater than 2 for other pharyngeal cancer (4).

Oral cancer screening programs aim to identify oral potentially malignant disorders (OPMDs) or early-stage oral cancer in patients who are generally asymptomatic, enabling the detection and early treatment of these lesions. In most studies and programs, this identification is achieved through a systematic visual inspection of the oral cavity and evaluation of the lymph node chains in the neck. Thus, patients identified with potentially malignant and malignant lesions can be provided with the necessary information and referrals to reduce the risk and/or complications resulting from the disease or clinical condition (5-7).

It is important to emphasize that the early diagnosis of oral cancer in initial stages, according to the TNM classification, has a significant impact on reducing the morbidity and mortality of the disease. In this regard, secondary prevention methods include accurate extraoral and intraoral examinations carried out by oral health professionals capable of recognizing clinically suspicious oral lesions, as most cases of oral cancer are preceded by OPMDs. For this reason, it is crucial for the dentist to be able to recognize these lesions and make appropriate referrals to a specialized professional. Similarly, the promotion of invitational and opportunistic screenings for oral cancer and measures to educate the population about identifying signs and symptoms of oral cancer and potentially malignant lesions during self-examination are important measures for secondary prevention. However, there still seem to be gaps in current knowledge that prevent the recommendation of including oral cancer as a screenable disease (8-10).

In this research work we performed an oral pathology evaluation in a high-risk population observed within an oral cancer screening developed in the southern region of Portugal. It was considered benign lesions/non-pathological conditions, oral potentially malignant disorders, and malignant lesions. The aims also include evaluate the possible relationship between the clinically observed lesions with sociodemographic variables, and tobacco or alcohol consumption.

## Material and Methods

This research took place during oral cancer opportunistic screening activities promoted by the non-profit organization Liga Portuguesa Contra o Cancro ‑ Núcleo Regional do Sul (LPCC-NRS). In 2022, a total of forty-eight oral cancer screening actions were conducted, and this research work includes data collected in forty-two actions, between March 19th and December 17th.

The location of the screening actions was defined according to the distribution of the LPCC-NRS support groups enfacing at-risk regions. Prior advertising was done online via the LPCC website and social networks, by physical adverts in the target region, and by healthcare facilities. Participation was accessible and available for any individual interested over 18 years old after written consent. Even so, the participation of minors was always carried out with the request and in the presence of their parents. However, preference was given for people with known risk factors: smokers aged 40 years or older; patients with complaints of pain or oral lesions (color/surface changes in the oral mucosa or with unusual increases in the size of oral structures); and patients referred by the medical doctor.

Out of the 3187 screened individuals in all the actions carried out in 2022, 2674 individuals were included in this research work. They were presented with a questionnaire followed by a visual examination and palpation of the oral and maxillofacial tissues. The questionnaire, provided in paper format, included a first part for collecting sociodemographic data, highlighting variables such as age, sex, population affinity, tobacco and alcohol habits, academic background, dental visit frequency, and time since the last dental appointment. The second part was filled out after a comprehensive visual examination of all structures of the oral cavity using a chair, a standard light source, compresses, and a disposable intraoral mirror for soft tissue retraction. Each action had two dentists present, both specialists in oral surgery, who jointly examined each individual and collected all observed anatomical changes and lesions, as well as the respective locations of each lesion.

The observed lesions were grouped into three distinct groups: benign lesions and non-pathological conditions; oral potentially malignant disorders (OPMDs); and suspicious malignant lesions. After the intraoral visual examination and recording of the lesions in the questionnaire, the results of each patient were registered and classified into three possible categories: 1 - no pre-neoplastic lesions or oral neoplasia; 2 - lesions with potential for malignant transformation; and 3 - suspected lesions of oral neoplasia. Individuals with positive results, classification 2 and/or 3, were referred to the National Health Service (NHS), through the oral cancer early intervention project, which includes dentists dedicated to oral medicine, to perform oral biopsies. Patients with malignant neoplasia histologically confirmed are then referred for hospital oncological treatment.

In a second analysis, all individuals classified as 2 and/or 3, according to the data collected by the LPCC-NRS, were contacted through a phone call between January and February 2023. They were questioned regarding the performance of a biopsy and its histopathological result.

The screened individuals' participation was voluntary. Furthermore, all individuals completed an informed consent for the treatment of personal data by LPCC-NRS and, in addition, they filled in the consent form for the possibility of being contacted for follow-up if necessary.

Participation in this questionnaire was voluntary and anonymous. For this work, the individual could participate in screening without participating in this questionnaire.

Statistical analysis was performed using the IBM SPSS Statistics® program version 28.0 (IBM, Armonk, NY, USA). In the descriptive analysis of the data, variables were described in terms of absolute and relative frequency. On the other hand, for the analytical analysis, associations between the variables under study were performed using the Chi-Square test or Fisher's exact test, depending on the type of variables being analyzed. Significance values where *p* < 0.05 were considered statistically significant.

## Results

**Figure 1:**
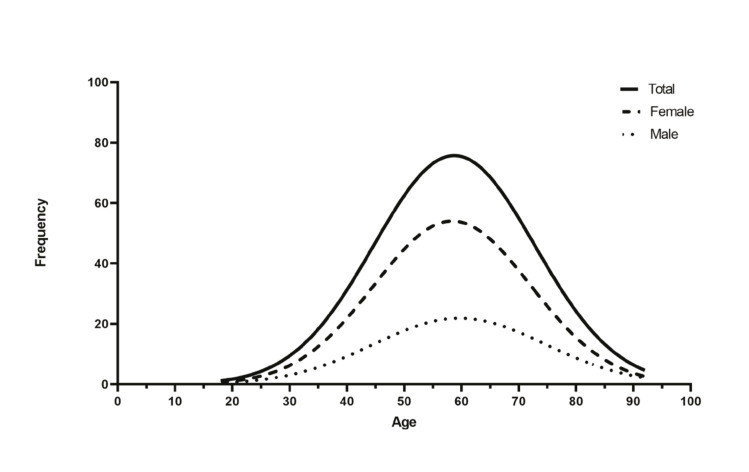
Representation of age distribution (Gaussian curves for the screened population).

Among the 2674 patients observed in the oral cancer screening actions included in this research, 815 (30.48%) were men, and 1859 (69.52%) were women. The mean age was 57 years (SD ± 14.29) with an age range from 9 to 92 years, as demonstrated in Fig. 1. Moreover, regarding population affinity, 2662 (99.55%) individuals were leucodermic, 11 (0.41%) were melanodermic, and 1 (0.04%) was xanthodermic. The remaining sociodemographic aspects and lifestyle characteristics are detailed in Table 1.

Out of 2674 participants, 2230 (83.40%) clinically presented alterations in the oral mucosa, while the remaining 444 (16.60%) did not show any changes. Considering that the same individual may have several lesions, which can belong to the same group or different groups of lesions, 2181 participants were diagnosed with benign lesions or non-pathological conditions, totaling 7435 diagnosed lesions. Additionally, 161 participants were diagnosed with oral potentially malignant disorders, accounting for a total of 179 lesions. Finally, 7 participants were diagnosed with suspicious malignant lesions. Absolute and relative frequencies of all 7621 observed lesions, analyzed in each group, are listed in Table 2.

Fordyce granules (16.30%) were the most common non-pathological condition, and hairy tongue (11.04%) was the most frequently observed benign lesion. Leukoplakia (43.58%) was the most common oral potentially malignant disorder. Seven lesions with a clinical diagnosis suggestive of oral squamous cell carcinoma were observed. The Table 3 provides all the information regarding distribution of age, sex, and population affinity of the 20 most common diagnoses.

**Figure 2:**
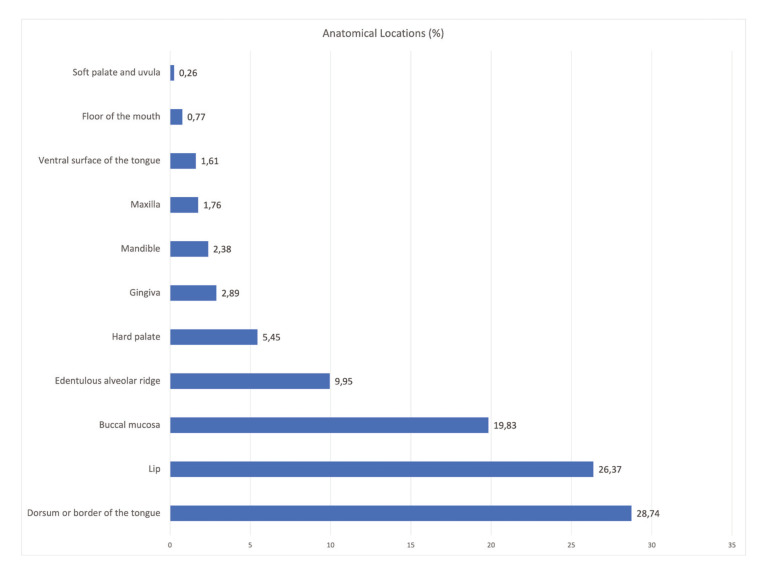
Relative frequencies (%) of the anatomical locations of all clinically diagnosed lesions.

Regarding the anatomical locations considered, 28.74% of the lesions occurred on the dorsum of the tongue, 26.37% on the lip, and 19.83% on the buccal mucosa. In 13.90% of cases, lesions occurred in two or more locations. The distribution of the anatomical locations of all diagnosed lesions appears in Fig. 2.

We analyzed possible relationships between oral potentially malignant disorders and malignant lesions with the smoking and alcohol consumption. The presence of leukoplakia is correlated with smoking habits (*p*=0.002), alcohol habits (*p*=0.003), and when both factors are synergistically present (*p*=0.006), influencing the development and appearance of the lesion, as shown in Table 4.

**Figure 3:**
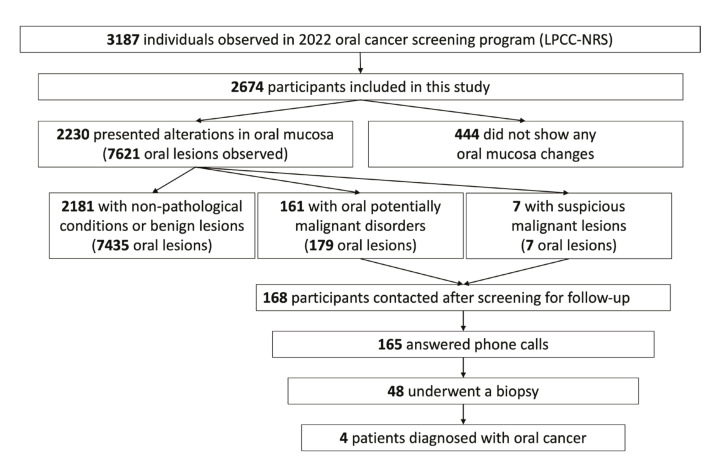
Outcomes of the oral screening program promoted by LPCC-NRS in 2022.

Additionally, a second analysis was conducted, including all participants identified as grade 2 - lesions with potential for malignant transformation (OPMDs) and/or grade 3 - suspected lesions of oral neoplasia. After a detailed analysis of the LPCC-NRS's database, the resulting 168 participants were contacted after screening for follow-up, including 161 identified with premalignant lesions and 7 participants with suspicious neoplasia. Only 165 participants answered phone calls and were questioned about undergoing a biopsy and its respective result. Among those questioned, 48 stated that they had undergone this diagnostic test. In total, 4 positive results for malignant neoplasms were identified, with one originating from a participant initially identified with a premalignant lesion and the rest from participants noted with suspected lesions of oral neoplasia. In Fig. 3 there is a flowchart with a summary of the outcomes of the screened population.

Of these 4 patients diagnosed with oral squamous cell carcinomas (OSCC), 3 are male, and 1 is female. Furthermore, all of them are over 49 years old and completed only basic education. Regarding smoking habits, 2 have been smokers for more than 30 years and the others are non-smokers. Concerning alcohol habits, 1 mentioned consuming alcoholic beverages daily for over 50 years, 2 occasionally consume alcoholic beverages for more than 30 years, and the other has never consumed alcohol. Lastly, 3 mentioned visiting the dentist less than once a year, and only one mentioned visiting one or more times per year. Concerning the location of the carcinomas, 2 are located on the lip, 1 on the floor of the mouth, and the other on the tongue.

## Discussion

This is the first study conducted in Portugal that combines the results obtained from invitation-based oral cancer screenings with such a vast geographical scope. Regarding the study sample, 2674 participants were observed throughout the year 2022, exceeding Monteiro *et al*. study, which involved 727 participants (11). When compared to other studies that included various oral cancer screening programs developed in Europe in the last decades, it can be noted that this sample size was larger than the ones found in the studies by Talamini *et al*. in Italy between 1991-1993, Vacher *et al*. in France in 1999, Downer *et al*. in 1994, Field *et al*. in 1995, Harris *et al*. between 1994-1999, Lim *et al*. in 2003, Nagao *et al*. between 1999-2000, and Scott *et al*. in 2010 in the United Kingdom (12-19).

In relation to the clinically diagnosed lesions, benign alterations and non-pathological conditions were the most frequent group of changes, present in 81.6% of participants. In addition, considering the three groups’ ages established, the majority of these alterations were diagnosed in the adult population (18-64 years). The most frequently found lesions in the geriatric population included oral candidiasis and hemangioma/vascular malformation.

Alternately, 168 of patients were identified as positive cases (6.3%), meaning they were clinically diagnosed with potentially malignant and/or malignant lesions. This percentage is slightly higher than the one obtained by Monteiro *et al*. and Lim *et al*., which were 3.3% and 4.2%, respectively, but quite close to the 5.7% obtained by Nunn *et al*. (11,17,20).

Considering the group of oral potentially malignant disorders, out of the 179 observed lesions, leukoplakia was the most frequent lesion, accounting for 43.58% of these lesions, followed by oral lichen planus or oral lichenoid lesions at 42.46%, actinic cheilitis at 12.29%, and proliferative verrucous leukoplakia at 1.68% of cases. Furthermore, there are several studies in which leukoplakia is also the most common OPMD, often followed by oral lichen planus (7,11,16,17,21,22).

It was also observed that the presence of leukoplakia is influenced by tobacco consumption (*p*=0.002), alcohol consumption (*p*=0.003), and when both habits coexist (*p*=0.006). In fact, it is known that among various possible risk factors, tobacco is closely related to the development of leukoplakia, with 71-90% of patients with leukoplakia being smokers (23). Additionally, the effect of alcohol consumption, as an isolated factor, remains contradictory, while this effect is higher when it acts synergistically with tobacco, increasing the risk of developing this oral potentially malignant disorder (24).

Based on clinical diagnosis, all the 168 patients classified with premalignant lesions or suspicious oral neoplasia, were identified and subsequently referred to the National Health Service (NHS), to perform an oral biopsy. After evaluating the results obtained, it was possible to confirm the diagnosis, with anatomopathological examination, in 48 patients, resulting in 44 oral potentially malignant disorders (OPMD) and 4 oral squamous cell carcinomas (OSCC).

Three main reasons may explain why only 48 of the 168 patients initially classified with premalignant lesions or suspicious oral neoplasia underwent biopsy on the second examination: cases of traumatic ulcers that had since healed; cases of frictional keratosis mistakenly identified as leukoplakia; and some cases of reticular lichen planus with typical lesions were not subject to histological confirmation.

When compared to other studies, the number of confirmed carcinomas is slightly higher than the 2 carcinomas found by Jullien *et al*. in the study conducted between 1994 and 1995 in the United Kingdom, Lim *et al*. in 2003 in the United Kingdom, and Monteiro *et al*. in 2011 in Portugal (11,17,25). Additionally, other studies such as those by Dombi *et al*. in 2001 in Hungary, Nagao *et al*., Nunn *et al*., and Scott *et al*. in the United Kingdom in 1999-2000, 2006-2008, and 2010, respectively, did not register any cases of oral cancer (18,19,21).

In this regard, it can be stated that these invitation-based oral cancer screening actions allowed for the positive identification of OPMDs and oral cancer. Furthermore, it is important to emphasize that the principle of screening is based on the assumption that malignancy, specifically most cases of oral cancer, is preceded by clinically evident lesions, which are well described in the literature. Thus, the primary objective of identifying OPMDs is the early detection of potential malignant transformation processes that allow for appropriate intervention, which should be based on the histopathological characteristics of the lesions (26).

According to various studies, visual examination of the oral cavity using good lighting, combined with palpation of the neck lymph nodes, has been the standard method described for identifying lesions in oral cancer screenings, allowing for the detection of relevant oral lesions with an accepTable degree of accuracy, making it a noTable screening test (5-7).

The need to determine the effectiveness of an oral cancer screening program, whether opportunistic or population-based, is of utmost importance, especially considering the high rates of morbidity and mortality associated with this disease, particularly in advanced stages (7). According to Warnakulasuriya *et al*. the strongest evidence demonstrating the effectiveness of a screening program and whether it is beneficial for the target population comes from well-designed and controlled studies that show that screened participants achieve better clinical outcomes resulting from early diagnosis compared to unscreened participants (6). Furthermore, it must be demonstrated that asymptomatic individuals with OPMDs or early-stage oral cancer have a significantly better response to treatment compared to individuals whose clinical situations are characterized by the presence of symptoms (6).

Additionally, up to the present day, no randomized controlled trials (RCTs) for oral cancer have been conducted in Europe, and the development and execution of these studies, applied to screenings, present several difficulties starting with the low incidence of oral cancer in most European countries. Therefore, in order to obtain statistically significant results in oral cancer screening actions, studies require a considerable sample size, consisting of hundreds of thousands of participants who can be systematically screened and controlled, and where a potential reduction in mortality of identified cases positive for oral cancer can be detected. Consequently, it is necessary to assess whether screening actions have demonstrated the ability to produce a positive effect on an intermediate outcome, such as early detection of malignant transformation processes associated with OPMDs (6). However, it is described that opportunistic screening of individuals, especially if they belong to high-risk groups, through routine visual examination performed by qualified healthcare professionals, may be effective and allow for better outcomes (7,17,24). In fact, the observation of 2674 patients led to the identification of more positive cases of oral cancer than expected, proportionally considering the standardized incidence rate for the Portuguese population, according to the National Oncology Register of 2019. These data indicate that screenings targeting high-risk populations are effective in identifying positive cases.

Considering the methodology applied in this study, the sample may not be representative of the general Portuguese population, given the circumstances in which the data were collected, affecting the generalization of the results. Furthermore, these early detection programs of oral cancer developed by LPCC-NRS aim to cover the most vulnerable population in the southern region of the country or those without access to primary healthcare and specialty consultations. However, this is the largest sample recorded in similar studies conducted in Portugal and one of the largest in Europe. Nevertheless, the results of this study should be interpreted in the context of potential methodological limitations. Regarding the recording of lesions, there are limitations inherent to the fact that the diagnosis is exclusively clinical, and some bias may have been introduced regarding the diagnosed lesions, although all participants were observed by two clinicians simultaneously. It is essential that all participants identified with OPMDs and suspected lesions of oral neoplasia are seen by a specialist and undergo biopsy to confirm the clinical diagnosis and thus receive appropriate treatment.

Additionally, further studies are needed to assess the inclusion of oral cancer more rigorously as a screenable disease in Portugal. Considering the Portuguese reality, the majority of oral cancer cases are diagnosed at advanced stages, often with regional metastases at the time of diagnosis, it is clear that the oral cancer screening developed by LPCC-NRS constitute important public health measures aimed not only at early diagnosis of oral cancer but also at increasing the survival rate of this cancer (11,27-29). Furthermore, the methodology adopted in this study can serve as a reference for the development of new studies and methodologies aimed at establishing campaigns and oral cancer screening programs for the Portuguese population.

## Conclusions

To the best of our knowledge, this represents the first study conducted in Portugal that combines the results of oral cancer screenings on such a large geographic scale, constituting the largest sample to date. Although benign pathology is the most frequently found, oral potentially malignant disorders and suspected malignant lesions were identified in 6.3% of participants. In addition, it was possible to histologically confirm the presence of 44 OPMDs and 4 OSCC. In this regard, we conclude that the oral cancer screening actions developed by LPCC-NRS were effective in identifying positive cases of premalignant lesions and oral cancer, using an intraoral visual examination of the oral cavity. Additionally, these activities also allowed to increase the population's knowledge and awareness of oral cancer, which potentially minimize the mortality and morbidity by early detection.

## Figures and Tables

**Table 1:** Characteristics of the population observed in oral cancer screenings (n=2674).

Variables		n (%)
Sex	Male	815 (30.48)
	Female	1859 (69.52)
Age	49 years	309 (11.56)
	49 years	2365 (88.44)
Population affinity	Leucodermic	2662 (99.55)
	Melanodermic	11 (0.41)
	Xanthodermic	1 (0.04)
Education	No education	33 (1.23)
	Primary school	854 (31.94)
	High school	982 (36.72)
	University	805 (30.10)
Tobaco comsumption	Non-smoker	1475 (55.16)
	Smoker	477 (17.84)
	Ex-smoker	722 (27.00)
Alcohol comsumption	Non-consumer	1162 (43.46)
	Consumer	1408 (52.66)
	Ex-consumer	104 (3.89)
Dentist visits	1x per year	1126 (42.11)
	1x per year	1548 (57.89)
Last dentistry appointment	Last 6 months	1134 (42.41)
	Between 6 months and 1 year	204 (7.63)
	1 to 2 years	503 (18.81)
	More than 2 years	610 (22.81)

**Table 2:** Absolute (n) and relative frequencies (%) of all 7621 observed lesions, analyzed in each group: benign lesions/non-pathological conditions, oral potentially malignant disorders, and malignant lesions.

Clinical diagnosis		n	%
Benign lesions and non-pathological conditions	Total Benign lesions and non-pathological conditions	7435	100
	Fordyce granules	1212	16.30
	Hairy tongue	821	11.04
	Fissured tongue	725	9.75
	Haemangioma/vascular malformation	707	9.51
	Frictional keratosis	701	9.43
	Physiological/racial melanic pigmentation	588	7.91
	Linea alba	553	7.44
	Torus/Exostosis	430	5.78
	Prosthetic stomatitis	312	4.20
	Traumatic fibroma (focal fibrous hyperplasia)	224	3.01
	Melanotic macule/melanocytic nevus	205	2.76
	Traumatic ulcer	194	2.61
	Benign migratory glossitis	113	1.52
	Smoker's melanosis	104	1.40
	Leucoedema	98	1.32
	Angular cheilitis	58	0.78
	Exfoliative cheilitis	58	0.78
	Amalgam tattoo	55	0.74
	Aphthous stomatitis	39	0.52
	Median rhomboid glossitis	36	0.48
	Inflammatory fibrous hyperplasia (prosthesis-induced)	33	0.44
	Herpetic lesions	30	0.40
	Mucocele	29	0.39
	Squamous papilloma	22	0.30
	Oral candidiasis	20	0.27
	Lingual varicosities	14	0.19
	Pyogenic granuloma	11	0.15
	Lipoma	11	0.15
	Other lesions	32	0.43
Oral potentially malignant disorders	Total Oral potentially malignant disorders	179	100
	Leucoplakia	78	43.58
	Oral lichen planus and oral lichenoid lesions	76	42.46
	Actinic cheilitis	22	12.29
	Proliferative verrucous leukoplakia	3	1.68
Malignant lesions	Oral squamous cell carcinoma	7	100

**Table 3:** Distribution of age, sex, and population affinity of the 20 most common clinical diagnoses.

Variables	nº	Age				Sex			Population affinity			
		0-17	18-64	≥ 65	(*p-value*)	Male	Female	(*p-value*)	Leucodermic	Melanodermic	Xanthodermic	(*p-value*)
Fordyce granules	1212	7 (0.58%)	832 (68.65%)	373 (30.78%)	(<0.001)	428 (35.31%)	784 (64.69%)	(<0.001)	1208 (99.67%)	3 (0.25%)	1 (0.08%)	(<0.001)
Hairy tongue	821	7 (0.85%)	525 (63.95%)	289 (35.20%)	(<0.001)	280 (34,10%)	541 (66.90%)	(<0.001)	818 (99.63%)	2 (0.24%)	1 (0.12%)	(<0.001)
Fissured tongue	725	3 (0.41%)	446 (61.51%)	276 (38.07%)	(<0.001)	233 (32.14%)	492 (67.86%)	(<0.001)	721 (99.45%)	3 (0.41%)	1 (0.14%)	(0.000)
Haemangioma/vascular malformation	707	1 (0.14%)	346 (48.94%)	360 (50.92%)	(<0.001)	230 (32.53%)	477 (67.47%)	(<0.001)	703 (99.43%)	3 (0.42%)	1 (0.14%)	(<0.001)
Frictional keratosis	701	6 (0.86%)	447 (63.77%)	249 (35.52%)	(<0.001)	267 (38.09%)	434 (61.91%)	(<0.001)	697 (99.43%)	3 (0.43%)	1 (0.14%)	(<0.001)
Physiological/racial melanic pigmentation	588	1 (0.17%)	412 (70.07%)	175 (29.76%)	(<0.001)	154 (26.19%)	434 (73.81%)	(<0.001)	581 (98.81%)	6 (1.02%)	1 (0.17%)	(<0.001)
Linea alba	553	9 (1.63%)	448 (81.01%)	96 (17.36%)	(<0.001)	164 (29.66%)	389 (70.34%)	(<0.001)	551 (99.64%)	1 (0.18%)	1 (0.18%)	(<0.001)
Torus/exostosis	430	2 (0.47%)	302 (70.23%)	126 (29.30%)	(<0.001)	148 (34.42%)	282 (65.58%)	(<0.001)	427 (99.30%)	3 (0.70%)	0 (0%)	(<0.001)
Prosthetic stomatitis	312	0 (0%)	146 (46.79%)	166 (53.21%)	(0.258)	56 (17.95%)	256 (82.05%)	(<0.001)	311 (99.68%)	1 (0.32%)	0 (0%)	(<0.001)
Traumatic fibroma (focal fibrous hyperplasia)	224	2 (0.89%)	129 (57.59%)	93 (41.52%)	(<0.001)	57 (24.45%)	167 (74.55%)	(<0.001)	222 (99.11%)	2 (0.89%)	0 (0%)	(<0.001)
Melanotic macule/melanocytic nevus	205	3 (1.46%)	132 (64.39%)	70 (34.15%)	(<0.001)	64 (31.22%)	141 (68.78%)	(<0.001)	205 (100%)	0 (0%)	0 (0%)	(-)
Traumatic ulcer	194	2 (1.03%)	110 (56.70%)	82 (42.27%)	(<0.001)	55 (28.35%)	139 (71.65%)	(<0.001)	194 (100%)	0 (0%)	0 (0%)	(-)
Benign migratory glossitis	113	3 (2.65%)	62 (54.87%)	48 (42.48%)	(<0.001)	40 (35.40%)	73 (64.60%)	(0.02)	113 (100%)	0 (100%)	0 (100%)	(<0.001)
Smoker's melanosis	104	0 (0%)	92 (88.46%)	12 (11.54%)	(<0.001)	34 (32.69%)	70 (67.31%)	(<0.001)	104 (100%)	0 (0%)	0 (0%)	(-)
Leucoedema	98	1 (1.02%)	62 (63.27%)	35 (35.71%)	(<0.001)	46 (46.94%)	52 (53.06%)	(0.544)	96 (97.96%)	2 (2.04%)	0 (0%)	(<0.001)
Leukoplakia	78	0 (0%)	40 (51.28%)	38 (48.72%)	(0.909)	44 (56.41%)	34 (43.59%)	(0.305)	77 (98.72%)	1 (1.28%)	0 (0%)	(<0.001)
Oral lichen planus and oral lichenoid lesions	76	0 (0%)	45 (59.21%)	31 (40.79%)	(0.09)	18 (20.68%)	58 (76.32%)	(<0.001)	76 (100%)	0 (0%)	0 (0%)	(-)
Angular cheilitis	58	0 (0%)	25 (43.10%)	33 (56.90%)	(0.294)	9 (15.52%)	49 (84.48%)	(<0.001)	58 (100%)	0 (0%)	0 (0%)	(-)
Exfoliative cheilitis	58	0 (0%)	32 (55.17%)	26 (44.83%)	(0.456)	28 (48.28%)	30 (51.72%)	(0.367)	58 (100%)	0 (0%)	0 (0%)	(-)
Amalgam tattoo	55	0 (0%)	31 (56.36%)	24 (43.64%)	(0.289)	24 (43.64%)	31 (56.36%)	(0.432)	55 (100%)	0 (0%)	0 (0%)	(-)

**Table 4:** Relationship between oral potentially malignant disorders and malignant lesions with smoking and alcohol habits.

Variables		nº	Tobaco				Alcohol				Smoking and alcohol consumpion		
		(%)	Non-smoker	Smoker	Ex-smoker	(*p-value*)	Non-consumer	Consumer	Ex-consumer	(*p-value*)	Does not smoke or drink	Smokes and drinks	(*p-value*)
Oral potentially malignant disorders (n=179)	Leukoplakia	78	29 (37.18%)	23 (29.49%)	26 (33.33%)	(0.002)	20 (25.64%)	52 (66.67%)	6 (7.69%)	(0.003)	64 (82.05%)	14 (14.95%)	(0.006)
	Oral lichen planus and oral lichenoid lesions	76	39 (51.32%)	19 (25.00%)	18 (23.68%)	(0.288)	42 (52.26%)	31 (40.79%)	3 (3.95%)	(0.155)	59 (77.63%)	17 (22.37%)	(0.288)
	Actinic cheilitis	22	12 (54.55%)	4 (18.18%)	6 (27.27%)	(0.998)	7 (31.82%)	13 (50.09%)	2 (9.09%)	(0.300)	18 (81.82%)	4 (18.18%)	(0.712)
	Proliferative verrucous leukoplakia	3 (1.68%)	1 (33.33%)	1 (33.33%)	1 (33.33%)	(0.423)	1 (33.33%)	2 (66.67%)	0 (0.0%)	-1.000	2 (66.67%)	1 (33.33%)	(0.927)
Malignant lesions (n=7)	Oral squamous cell carcinoma	7 (100%)	2 (28.57%)	4 (57.14%)	1 (14.29%)	(0.143)	2 (28.57%)	5 (71.43%)	0 (0.0%)	(0.340)	3 (42.86%)	4 (57.14%)	(0.557)

## Data Availability

The datasets used and/or analyzed during the current study are available at .

## References

[1] Warnakulasuriya S (2009). Global epidemiology of oral and oropharyngeal cancer. Oral Oncol.

[2] Muller S, Tilakaratne WM (2022). Update from the 5th Edition of the World Health Oraganization Classification of Head and Neck Tumors: Tumours of the Oral Cavity and Mobile Tongue. Head Neck Pathol.

[3] Sung H, Ferlay J, Siegel RL, Laversanne M, Soerjomataram I, Jemal A (2021). Global Cancer Statistics 2020: GLOBOCAN Estimates of Incidence and Mortality Worldwide for 36 Cancers in 185 Countries. CA Cancer J Clin.

[4] Cunha AM, Compton K, Xu R, Mishra R, Drangsholt MT, Antunes JLF (2023). The Global, Regional, and National Burden of Adult Lip, Oral, and Pharyngeal Cancer in 204 Countries and Territories: A Systematic Analysis for the Global Burden of Disease Study 2019. JAMA Oncol.

[5] Warnakulasuriya S, Kerr AR (2021). Oral Cancer Screening: Past, Present, and Future. J Dent Res.

[6] Warnakulasuriya S, Fennell N, Diz P, Seoane J, Rapidis A, Programme LLL (2015). An appraisal of oral cancer and pre-cancer screening programmes in Europe: a systematic review. J Oral Pathol Med.

[7] Brocklehurst P, Kujan O, O'Malley LA, Ogden G, Shepherd S, Glenny AM (2013). Screening programmes for the early detection and prevention of oral cancer. Cochrane Database Syst Rev.

[8] Ogden G, Lewthwaite R, Shepherd SD (2013). Early detection of oral cancer: how do I ensure I don't miss a tumour?. Dent Update.

[9] Nikitakis NG, Pentenero M, Georgaki M, Poh CF, Peterson DE, Edwards P (2018). Molecular markers associated with development and progression of potentially premalignant oral epithelial lesions: Current knowledge and future implications. Oral Surg Oral Med Oral Pathol Oral Radiol.

[10] Sarode SC, Sarode GS, Karmarkar S (2012). Early detection of oral cancer: detector lies within. Oral Oncol.

[11] Monteiro LS, Salazar F, Pacheco JJ, Martins M, Warnakulasuriya S (2015). Outcomes of invitational and opportunistic oral cancer screening initiatives in Oporto, Portugal. J Oral Pathol Med.

[12] Talamini R, Barzan L, Franceschi S, Caruso G, Gasparin A, Comoretto R (1994). Determinants of compliance with an early detection programme for cancer of the head and neck in north-eastern Italy. Eur J Cancer B Oral Oncol.

[13] Vacher C, Legens M, Rueff B, Lezy JP (1999). [Screening of cancerous and precancerous lesions of the oral mucosa in an at-risk population]. Rev Stomatol Chir Maxillofac.

[14] Downer MC, Evans AW, Hughes Hallet CM, Jullien JA, Speight PM, Zakrzewska JM (1995). Evaluation of screening for oral cancer and precancer in a company headquarters. Community Dent Oral Epidemiol.

[15] Field EA, Morrison T, Darling AE, Parr TA, Zakrzewska JM (1995). Oral mucosal screening as an integral part of routine dental care. Br Dent J.

[16] Harris CK, Warnakulasuriya KA, Cooper DJ, Peters TJ, Gelbier S (2004). Prevalence of oral mucosal lesions in alcohol misusers in south London. J Oral Pathol Med.

[17] Lim K, Moles DR, Downer MC, Speight PM (2003). Opportunistic screening for oral cancer and precancer in general dental practice: results of a demonstration study. Br Dent J.

[18] Nagao T, Warnakulasuriya S, Gelbier S, Yuasa H, Tsuboi S, Nakagaki H (2003). Oral pre-cancer and the associated risk factors among industrial workers in Japan's overseas enterprises in the UK. J Oral Pathol Med.

[19] Scott SE, Rizvi K, Grunfeld EA, McGurk M (2010). Pilot study to estimate the accuracy of mouth self-examination in an at-risk group. Head Neck.

[20] Nunn H, Lalli A, Fortune F, Croucher R (2009). Oral cancer screening in the Bangladeshi community of Tower Hamlets: a social model. Br J Cancer.

[21] Dombi C, Vörös-Balog T, Czeglédy A, Hermann P, Vincze N, Bánóczy J (2001). Risk group assessment of oral precancer attached to X-ray lung-screening examinations. Community Dent Oral Epidemiol.

[22] Warnakulasuriya S, Kujan O, Aguirre-Urizar JM, Bagan JV, González-Moles M, Kerr AR (2021). Oral potentially malignant disorders: A consensus report from an international seminar on nomenclature and classification, convened by the WHO Collaborating Centre for Oral Cancer. Oral Dis.

[24] Speight PM, Epstein J, Kujan O, Lingen MW, Nagao T, Ranganathan K (2017). Screening for oral cancer - a perspective from the Global Oral Cancer Forum. Oral Surg Oral Med Oral Pathol Oral Radiol.

[25] Jullien JA, Downer MC, Zakrzewska JM, Speight PM (1995). Evaluation of a screening test for the early detection of oral cancer and precancer. Community Dent Health.

[26] Holmstrup P, Vedtofte P, Reibel J, Stoltze K (2007). Oral premalignant lesions: is a biopsy reliable?. J Oral Pathol Med.

[27] Monteiro LS, Antunes L, Bento MJ, Warnakulasuriya S (2013). Incidence rates and trends of lip, oral and oro-pharyngeal cancers in Portugal. J Oral Pathol Med.

[28] Albuquerque RP, López-López J, Jané-Salas E, Rosa-Santos J, Ibrahim C (2012). A pioneering epidemiological study investigating the incidence of squamous cell carcinoma of tongue in a Portuguese population. Med Oral Patol Oral Cir Bucal.

[29] Monteiro LS, Amaral JB, Vizcaíno JR, Lopes CA, Torres FO (2014). A clinical-pathological and survival study of oral squamous cell carcinomas from a population of the North of Portugal. Med Oral Patol Oral Cir Bucal.

[23] Speight PM, Epstein J, Kujan O, Lingen MW, Nagao T, Ranganathan K (2017). Screening for oral cancer - a perspective from the Global Oral Cancer Forum. Oral Surg Oral Med Oral Pathol Oral Radiol.

